# How the Custom Suppresses the Endowment Effect: Exchange Paradigm in Kanak Country

**DOI:** 10.3389/fpsyg.2021.785721

**Published:** 2022-01-25

**Authors:** Jean Baratgin, Patrice Godin, Frank Jamet

**Affiliations:** ^1^Université Paris 8, Laboratoire Cognition Humaine et Artificielle, Saint-Denis, France; ^2^Probability, Assessment, Reasoning and Inferences Studies Association, Paris, France; ^3^Université de la Nouvelle Calédonie, Laboratoire TROCA, Nouméa, France; ^4^CY Cergy Paris Université, Paris, France

**Keywords:** decision-making, endowment effect, exchange paradigm, Kanak culture, custom, pragmatics, human interaction, politeness social norms

## Abstract

In this paper, Knetsch's exchange paradigm is analyzed from the perspective of pragmatics and social norms. In this paradigm the participant, at the beginning of the experiment, receives an object from the experimenter and at the end, the same experimenter offers to exchange the received object for an equivalent object. The observed refusal to exchange is called the endowment effect. We argue that this effect comes from an implicature made by the participant about the experimenter's own expectations. The participant perceives the received item as a gift, or as a present, from the experimenter that cannot be exchanged as stipulated by the social norms of western politeness common to both the experimenter and the participant. This implicature, however, should not be produced by participants from Kanak culture for whom the perceived gift of a good will be interpreted as a first act of exchange based on gift and counter-gift. This exchange is a natural, frequent, balanced, and indispensable act for all Kanak social bonds whether private or public. Kanak people also know the French social norms that they apply in their interactions with French people living in New Caledonia. In our experiment, we show that when the exchange paradigm takes place in a French context, with a French experimenter and in French, the Kanak participant is subject to the endowment effect in the same way as a French participant. On the other hand, when the paradigm is carried out in a Kanak context, with a Kanak experimenter and in the vernacular language, or in a Kanak context that approaches the ceremonial of the custom, the endowment effect is no longer observed. The same number of Kanak participants accept or refuse to exchange the endowed item. These results, in addition to providing a new explanation for the endowment effect, highlight the great flexibility of decisions according to social-cultural context.

## 1. Introduction

For more than 40 years, numerous studies have shown that people seem to value a good they possess more than an equivalent good they do not possess. This *endowment effect* (Thaler, 1980) is observed in particular in the economic and psychological literature through the *Exchange Paradigm* (from now on designated as *EP*). The *EP* consists of two stages: The experimenter first checks whether a group of participants are indifferent to possessing an object *A* or to possessing an object *B*. Then, the remaining participants in the study are randomly endowed with one of the two equivalent goods *A* and *B* and after a time (in which the participant does some work or exercise) are then given the opportunity to exchange the endowed good for the other good that they did not receive. The endowment effect is reflected in a refusal to exchange the received good.

This refusal to exchange is observed in young children (Harbaugh et al., 2001; Horowitz and McConnell, 2002; Lucas et al., 2008; Da Silva et al., 2014) and also in certain non-human primates (Lakshminaryanan et al., 2008; Kanngiesser et al., 2011; Brosnan et al., 2012; Flemming et al., 2012, to cite a few). It is considered a rationality bias with respect to the prescription of standard economic theory which states that individuals' preferences over goods are independent of whether or not they posses it. In *EP*, the endowment of one of the two goods should not change the initial lack of preference of individuals between the two goods. Thus, if an individual has no preference between good *A* and good *B* and they own one of them, they should have no preference between keeping it or trading it. In *EP*, we should find a similar proportion of participants agreeing or refusing to exchange the received good for the other when they initially have no preference between the two objects. The endowment effect reflects this “exchange asymmetry” (Plott and Zeiler, 2007; Marzilli Ericson and Fuster, 2014) in favor of the given good.

The traditional explanation of this behavior is given by prospect theory and in particular by the notion of *loss aversion* (Kahneman et al., 1990; Tversky and Kahneman, 1991). Psychologically, a gain triggers a feeling of satisfaction, a loss triggers a feeling of dissatisfaction. Comparing the satisfaction level for a gain of a value *X* to the level of dissatisfaction triggered by the loss of a value of −*X* we observe that the level of dissatisfaction is higher than that of the satisfaction (Thaler, 1980; Knetsch, 1989; Kahneman et al., 1990). According to some authors, loss aversion would be the result of an evolutionary process based on the importance of overvaluing these goods when bargaining to acquire more resources. This would allow the maintenance of more offspring than people who correctly value (or undervalue) what they possess (Heifetz and Segev, 2004; Huck et al., 2005). This would be caused by an ancestral impulse to “defend one's own territory.” Authors suggest there might be a genetic predisposition to acquire and to preserve goods (food, shelters, tools and territory) which allows the individual and collective survival (Stake, 2004; Gintis, 2007)^1^. An alternative explanation of a psychological nature is given with the singular status of the feeling of ownership of an object. The endowment effect would be caused by a simple possession effect which would favor a strong attachment and a privileged relationship with the object (see for a review Morewedge and Giblin, 2015).

To our knowledge, in the literature, few studies have emphasized that *EP* involves a particular interaction between two protagonists. In a study on children 4 years old, Lucas et al. (2008) found a link between the endowment effect and success in the theory of mind (TOM) task. It is thus possible that there is a link between the endowment effect and the participant's interpretation of the experimenter's intentions and beliefs. In order to understand participants' interpretations, we must first understand what are these interactions. In *EP*, an experimenter hands out an object and a recipient receives it. The experimenter is very often a teacher and the recipients are students. The relationship governing the interaction between them is hierarchical, it is unbalanced. The social status of the two people is not the same. The first phase of interaction is the experimenter's endowment to the participants. This interaction certainly has a strong contextual effect. It is rare for a professor to give something to their students in a university course and this certainly surprises the participants. Moreover, we can observe that all participants accept this gift. The second phase of interaction is the request for an exchange. This request is also unusual, at least in Western customs. Without making a pragmatic analysis of the task, Plott and Zeiler (2007); Klass and Zeiler (2013) hypothesize that the participants consider the object given to them “is a gift from the experimenter, even though the experimenter might simply intend to convey that subjects now own [the object]” (Plott and Zeiler, 2007, p. 1454). This interpretation of the endowed object as a present given by the experimenter would lead them to prefer this object to another and to feel obliged to refuse the exchange. They observe an absence of endowment effect when the endowed object is not physically given (see also Knetsch and Wong, 2009) or is given randomly in concordance with the other object. In turn, they observe a strong endowment effect when the experimenter explicitly gives the object as a gift. Many of the results in the literature can be explained using this interpretation.

In *EP*, the endowment effect also seems to disappear when the goods given and exchanged are goods of exchange, such as money (Svirsky, 2014). Moreover, individuals experienced in markets seem less subject to the endowment effect (List, 2003; Engelmann and Hollard, 2010). However, exchange goods such as money are not usually used as gifts, and market professionals who are used to receiving goods and reselling them are certainly less inclined to consider the endowment as a present. Finally, in the only study we know of using *EP* with a non-Western population, Apicella et al. (2014) show that members of hunter-gatherer tribes (Hadza Bushmen of Northern Tanzania) who are highly exposed to modern society and markets are reluctant to trade in *EP*. Hadza with little exposure to modern society and markets do not show the same reluctance to trade. It is possible to analyze these observed differences as a different social response to receiving a gift from the Western experimenter^2^. The Hadza who have experience of Western social norms would behave like Westerners when they receive a gift the Hadza who are not familiar with the customs of the Western world would behave according to their own social norms by exchanging and sharing it with the rest of the tribe (see for example Marlowe, 2004). Thus, we support the hypothesis of Plott and Zeiler (2007) that *EP* would encourage participants to represent the question of exchange in terms of whether or not to exchange a gift received.

As noted by Horak (2018), the vast majority of cross-cultural economic experiments provide evidence that culture can moderate economic behavior, but few studies explain the reasons for this behavioral difference across societies in the field of decision-making. We argue that culture, through the social norms of individuals, shapes representations and implicatures which differ on the expectations of the experimenter, but that the underlying cognitive constructs are the same for all individuals. More generally, *EP* illustrates singular situations of interaction between the experimenter and the participant which are giving and exchanging. Such a paradigm is not only interesting for psychologists and economists but also represents an emblematic paradigm for anthropologists. Indeed *EP* illustrates experimentally the anthropological paradigm of the “gift” with its triptic “giving,” “receiving,” and “giving back” (discussed since Mauss, 1924; Malinowski, 2018) which can be easily studied in different cultures. Indeed, Mauss (1924) in his study of Maori gift giving (the “spirit of the gift”) suggests that, in many societies, the objects given are ultimately “inalienable,” that is, they cannot be entirely detached from the giver, but carry within them something of the personality of that person. They carry significant implications for the future actions of the receiver and for the relationship between them. This originality of Melanesian societies was observed by Henrich et al. (2005) in a study of the ultimatum game^3^ among the *Au* and *Gnau* of Papua New Guinea in which the majority of participants reject the seemingly generous offers of the proposer when it represents more than 50%. The explanation provided by Henrich et al. (2005) is as follows:

In these societies, accepting gifts, even unsolicited ones, implies a strong obligation to reciprocate at some future time. Unrepaid debts accumulate, and place the receiver in a subordinate status. Further, the giver may demand repayment at times or in forms (e.g., political alliances) not to the receiver's liking, but the receiver is still strongly obliged to respond. As a consequence, excessively large gifts, especially unsolicited ones, will frequently be refused. (Henrich et al., 2005, p. 811).

This specificity of gift and return-gift (reciprocity norm), as a key factor in social interactions, present in all Melanesian societies (Godin, 2015; Tcherkézoff, 2016), certainly produces a different interpretation of the experimenter's gift than that of the Western participants gift. In an *EP* effected in a Melanesian context, a Melanesian participant who receives an object from a Melanesian experimenter will also tend to perceive this endowment as a gift. However, this gift is not perceived as “a present” (as it is for Westerners) but as the source of the exchange that allows the interaction between the two individuals (here the experimenter and the participant) to come alive. It will be the same later in the question of exchange with an equivalent object, the participant can either accept it or refuse the exchange. We assume that there is no endowment effect in *EP* if the interaction involves two Melanesian individuals.

The Kanak of New Caledonia in the South Pacific is a native Melanesiane population located for the most part in the Northern Province and in the province of the Loyalty Islands. Kanak society has several levels of customary authority, from the 4,000 or 5,000 family clans to the eight customary areas that make up the territory. At the head of clans are clan chiefs and clans constitute 341 tribes, with a chief at the head of each one. The tribes are then grouped into 57 customary chiefdoms, with chiefs at their head, and forming the administrative subdivisions of the customary areas. In Kanak society, giving and exchanging play a primordial role in private interactions but also in the structure of society, notably with the ceremony of the custom. The Kanak population is all the more interesting to study because it is partially bicultural. Thus, the Kanak are also perfectly familiar with all the social codes of French Western society (notably through the obligation for all French children from the age of 3 to attend the school of the French Republic). The cross-cultural studies on Kanak populations are rare, however we can note Jamet et al. (2014).

After a pragmatic analysis of the possible implicatures of Western participants confronted with *EP*, we will explain the particularity of Kanak society and why Kanak participants should not be subjected to the endowment effect in *EP*. In our experiment we will distinguish the French Western context and the Kanak context in order to look at the adaptive capacity of the Kanak between the two cultures.

## 2. The Ambiguity of the Exchange Question

The literature on reasoning and decision making offers numerous examples in which behaviors or responses given by participants, initially judged to be erroneous, reveal a coherence with respect to the inferred representation of the participants to the requested task. These representations can be explained by the different pragmatic implicatures coming from the violations of the conversational maxims of cooperation of Grice (1975) (see Dulany and Hilton, 1991; Schwarz et al., 1991; Sperber et al., 1995; Baratgin and Noveck, 2000; Macchi, 2000; Politzer and Macchi, 2000; Baratgin, 2002, 2009; Bagassi and Macchi, 2006; Baratgin and Politzer, 2006, 2007, 2010; Macchi and Bagassi, 2012; Politzer, 2016; Macchi et al., 2019, 2020; Bagassi et al., 2020; Baratgin et al., 2020, for examples). The experimental paradigms are constructed through speech acts and the gestures of the experimenter and are, as in any communication fact, pragmatic in nature (Sperber and Wilson, 1986, 2002). The participants make assumptions about the experimenter's expectations and use the simplest interpretation procedure which consists in inferring from the communicative stimulus the intention most relevant to their own point of view Sperber (1994). However, what is relevant to the participant may be different from what the experimenter actually intends to communicate. Questions are relevant when they lead the person to whom they are directed to respond in a relevant way (i.e., questions that require the least cognitive cost for the most contextual effect). The exchange question in *EP*, can be interpreted differently depending on the social norms of the participants. In particular, we will be interested in the different interpretations of the question of exchange in a Western population (French population of Metropolitan France) and in a Kanak population in the South Pacific. The issue of social norms in the act of giving is important to understand. Before looking specifically at the issues of gift and exchange in French and Kanak society, we will briefly review the various theories of social norms of politeness.

In social interactions, norms of politeness are crucial. They help reinforce collaboration through rules that are known to all members of society. These rules lead to implicit expectations in actions and words from people interacting together (Geraci, 2020; Geraci and Franchin, 2021; Geraci et al., 2021). Since Leech (1983), it is admitted that communicative exchanges are subjected to the fundamental postulate “to be polite” in our interactions. He proposes a series of maxims and sub-maxims based on the “cost” and the “benefit” in relation to the interaction between *self* and *other*. Thus, if the benefit is higher to the receiver than the cost, then it seems to be more polite. On the contrary, if the cost is higher than the benefit to the recipient, then it seems to be less polite.

The model of politeness theory described by Brown and Levinson (1987) is the reference for most studies on politeness (for a critique see Fraser, 1990; Watts, 2003). The model is also intended to be “universal.”^4^ The authors use the notions of *face* from (Goffman, 1967, 1971) to define their pragmatic theory of politeness. The *face* is: “the positive social value a person effectively claims for himself by the line others assume he has taken during a particular contact” (Goffman, 1967, p. 267). Brown and Levinson (1987) maintain that each individual has two types of *face*; a negative face and a positive face. The positive face is the individual's desire to be appreciated and approved in their social interaction (valuing the image of the individual) and the negative face is their desire for freedom of action and to impose their ideas and desires (protection or defense of “[their] territory”). Brown and Levinson (1978, 1987), also use the hypothesis of Goffman (1967) that, for an individual, any interaction with others is a potential source of conflict. The rules of politeness correspond to ritual constraints, codes and strategies which aim at preserving the face (positive or negative for Brown and Levinson, 1987) of the interlocutors by avoiding conflicts. These norms thus assume the essential functions of facilitation and regulation of social interactions and are, following a learning phase at the youngest age, strongly internalized (Talwar et al., 2007). Throughout the course of the interaction, the interlocutors perform a number of verbal or non-verbal acts that potentially constitute threats to the positive or negative face of both interlocutors. The objective for the participants is to reduce as much as possible these Face Threatening Acts (FTA).

### 2.1. The Question of Exchange of Gifts in the Western Culture

In French (but also in all European languages), “donner” (to give) and “échanger” (to exchange) are the two verbs used to translate the action of transferring goods and services. The verb “donner” is polysemous, it is used when there is no financial counterpart (“Je donne àune association charitative,” I make a donation to a charity) or with a financial counterpart (“Donnez-moi un kilo de pomme,” Give me a kilo of apples, at a shop against money). It is also used for non-material goods (saying hello by shaking hands is called “donner une poignée de main,” giving a handshake). The verb *to exchange* is less ambiguous. It means to give something up in return for something else. We understand that the exchange is conditional to the counterpart. The counterpart comes to fill the imbalance, to cancel the loss. It is at the same time: the mean of the exchange, required, mandatory and a sign of the end of the exchange. To be exact, we will also use this verb by analogy with the exchange of material goods for the exchange of “words” or “politeness” (Testart, 1997; Darmangeat, 2016). In *EP*, when the experimenter gives an object to the participants, the context is not that of an experimental context. The situation takes place in a class in which the experimenter is the teacher and in which they offer an object to their students (who do not explicitly know that they are participating in an experiment). Thus, when they say, without any justification or explanation: “I give you a mug,” the student may be surprised by this gift. He does not know if this gift is free (or at least seems to be) or if the teacher will later ask for something in return^5^. One can therefore really consider, as do Plott and Zeiler (2007), that in this first important stage of *EP* the given object corresponds to, even if it is not explicitly specified, an item given without counterpart: a gift. Thus, for the participant, this is a new, free, altruistic and generous action. The object given is interpreted as a present. According to Brown and Levinson (1987) such an offer should be understood as an FTA against the negative face of the participant, since the acceptance sets up for them a kind of debt which they will have to pay back. However, we think that this gift can also be seen as an act that enhances the positive face of the participant, especially since this gift is provided by the experimenter (their teacher) who is hierarchically superior. Thus, this offer should be understood by the participant as a friendly sign of sympathy and of appreciation from the experimenter; as a gift to be accepted. One can think then that for Western participants, in an automatic way, the social norms of politeness acquired in society inherent to the reception of a gift are activated. The participants accept this gift which reinforces a pragmatic collaboration with the experimenter since in our contemporary western societies, the act of giving something is perceived by the participant as a significant act of sympathy which expresses a particular link between giver and receiver. This act of giving is therefore received positively by the participant. If the given object is perceived as a present, the participants must follow the socially appropriate behavior in accepting the object and also expect the experimenter to follow the socially appropriate behavior of the giver. This is what the experimenter does at first, as they no longer talk about the given object and move on. Often they continue their lecture or the students are tasked to do some work (e.g., to fill in a short questionnaire, see Knetsch, 1989). The second stage of *EP*, that of the request for an exchange, clearly corresponds to a departure from the norm of politeness expected of someone offering a gift. This violation of the social norms is especially strong when decision-making (accepting or refusing the exchange) happens face-to-face with the experimenter. This violation of the social norms of politeness is all the stronger contextually. In French there is a well-known expression dedicated to the situation: “donner c'est donner et reprendre c'est voler” (giving is giving and taking back is stealing). This request for an exchange, still without any justification or explanation, for the object previously “offered” by the participant encourages several possible implicatures on the part of the participant.

- The experimenter regrets the first present they gave and now offers another object to replace the initial gift or to get it back (though it is not because they do not think it is important enough as the object offered in return has a similar value).
- The first endowment was in fact not really a free gift but that it served for this question of exchange.

In the first case, this request is seen as a threat to the negative face, to take back an object that is part of my “territory.” Moreover, if the participant accepts the exchange, then they in turn produce a threat on the positive face of the experimenter by explicitly showing that they did not in fact appreciate the gift and are happy to exchange it. Thus, the participant should not exchange because that would be to say that the first gift was not satisfactory. In the second case, the context is updated by the participant. The initial offer was clearly an FTA and required something to be given in return. There is obviously a violation in the cooperation with the giver and on the receiver's side, the cooperation is also stopped by refusing the exchange. In either case the appropriate response is to keep the object. It is this response that requires the least cognitive effort and allows one to remain in the initial adequate context of the representation of the item as a gift. Thus, in our view, *EP* implicitly favors a response that conforms to the norms of politeness in use in Western societies. This explanation was supported by the disambiguation of the paradigm using a NAO robot instead of a human experimenter. In this disambiguated context, where the norms of politeness are not activated (one is not polite with a machine), the endowment effect disappears (see Masson et al., 2015, 2016; Masson et al., 2017a,b).

### 2.2. The Question of Exchange of Gifts in the Kanak Culture

To understand how the question of exchange in *EP* should be interpreted by Kanak participants, we must first explain how exchange structures social relations in Kanak society. In order to do this, we must make what is a complete shift of reference frame for a Westerner. What allows society to exist in the Western world is the democratic (individualistic) relationship. All the aspirations, all the new rights, all the reductions of inequality of the citizens are discussed and settled in the framework of the democratic game. Once the law is voted by representatives, it applies to all and by all. Laws are followed because they have been voted in the name of the people, by the people's representatives. All these laws constitutes the social contract. It will evolve as society changes. Western society is a law-based society. Kanak society is a strongly holistic society (Dumont, 1983; Godin, 2015; Tcherkézoff, 2016) established on another principle than the individual rights. This first principle is exchange, more precisely of exchange of gifts. The exchange is what allows the Kanak society to make society. In order to understand exchange in the Kanak world, we will begin by briefly examining the notion of exchange from a linguistic point of view in Kanak languages in relation to French, and we will then present custom, a structuring practice in Kanak society.

This importance of exchange is visible from a linguistic point of view different terms are used to specify the different types of exchange. For example, in the *némi* language, the generic term to express exchange is *pe-na-aman*, literally “to give [na] things [aman] to each other [pe].” This is the dedicated name attributed to a reciprocal giving of gifts and not to a simple isolated transfer. In this term, the action is double, it integrates the “gift,” but also “the gift in return” (*hiwec*). This double action has for main objective not to satisfy a material balance of the exchange but *to tie, to support and to maintain the social bonds* (social bonds of kinship, of neighborhood, of mutual aid...). A second term *ge-na-aman* is used for “ceremonial” exchanges. These are the exchanges that sanction the different stages of a person's social life, from birth to death. These exchanges of gifts must take place between the paternal and maternal relatives of the person and, beyond a desire to honor the person themselves, proceeds from a whole ritual cycle of renewal of “life” (named *maric*) (Godin, 2018). The prototypical *pe-na-aman* exchange of gifts is manifested by a double transfer of objects: a transfer from the individual (or group) X to the individual (or group) Y then a reciprocal transfer of the individual (or of the group) Y toward the individual (or toward the group) X. Contrary to the idea that the gift is a free act performed without any hope of a return, the first of the two transfers is here accomplished in expectation of a return, but here unlike what happens in the exchange this return is not conceived or perceived as a equivalent or as a compensation, but as a sign of reciprocity (Komter, 2007) and an acquiescence in the social relationship that one wants to create or continue. In Kanak society as in many other Oceania societies, the reciprocity of gifts establishes the social bond. And from this point of view, the value of objects is less important here than their social significance, even if there is indeed an accounting of given and returned “things” which shows the partners' concern to live up to the level of the established link and of reciprocal obligations that it imposes (Godin, 2015, 2018).

This dyadic form of the exchange of gifts is found in all welcoming ceremonies. People arriving face the hosts and the latter proceed to a gift of goods and words toward the welcomed. Then it is the welcomed person who proceeds to a return-gift toward the welcoming person. The conclusion of this face-to-face meeting ends with a meal (Monnerie, 2020). This modality of exchange is called “coutume,” French for custom. In Kanak culture, all the events (birth, marriage, death, adoption of a child, dispute between neighbors, visit to a friend, etc.) that punctuate life, whether they are large or small, are all occasions for “faire un geste coutumier” (making a customary gesture). Making the custom consists of carrying out a set of acts that are indispensable for entering the Kanak world and to create or reestablish social ties in this world (Godin, 2015; Monnerie, 2017, 2020; Bretteville, 2019). It gathers a whole set of gestures in a space of speech and listening. Seen from the outside, its specificity is that it is based both on an exchange of goods and on an exchange of words. It should always be kept in mind that for the Kanak person, speech and gifts are inseparable. Relationships are considered to be unpredictable and ever changing and thus must be periodically reactivated. The relationship always precedes the things exchanged. These exchanges involve both the living and the dead. This system of social relations is based on a deep respect for natural forces, for the power of the word and for the gesture of exchange (see for more details Godin, 2015). The custom can be carried out between two people, between two clans, between two tribes, between two districts between customary areas and then mobilizes hundreds of participants. The custom allows each of the protagonists to live the double experience of being the host and the welcomed. This experience makes it possible to understand the relative character of these positions.

The Kanak person is thus confronted throughout their life in a daily manner with the action of giving and return-giving. We believe that when confronted with *EP*, the Kanak participant may have a different representation of the task, all the more so if the experimenter (the donor) is also Kanak and speaks to them in their language. Thus, in this situation, the gift is taken as an initial exchange that must be followed at some point, immediately or another day, by a return gift from the participant to the experimenter. We think that the gift made by the experimenter will be interpreted differently depending on whether they do or do not belong to Kanak culture. The differences will only really appear when offering to exchange the first gift for another. If the experimenter is a foreigner, European or other, the exchange proposal will be interpreted in the register of the material exchange. If the experimenter is Kanak, on the contrary, it will be understood in the register of the gift which subordinates the material value of things to the establishment of a personal relationship. The exchange can then be carried out with all the less reluctance since it can be perceived as an extension of the gift, or even its reiteration. The existence of a family bond between the experimenter and the participant will only reinforce this tendency to accept the exchange.

The aim is always to make connections. Thus, there is no surprise in receiving something, even if it is a teacher, because it is a usual action in any human relationship in the social norm of the Kanak culture. When it comes to the question of exchange there is here, contrary to the Western context, no departure from the norm. We remain in the continuation of the previous exchange. It is not at all unusual for the Kanak participant that someone who offered something asks to exchange the good that has just been given. The desire for a link between individuals is thus pursued. Thus, the participant may accept the exchange or refuse it for a future exchange with another object. Thus, our first hypothesis is that in this situation of an interaction between a Kanak experimenter and Kanak participants (“Kanak” context), we should not have an endowment effect. Now when the experimenter is Western in a Western context, it is possible that in this “French” context the Kanak participant, who has both French and Kanak culture, adopts the social norms of French politeness and behaves like a French participant with an endowment effect. Finally, we hypothesize that the simple interaction with an experimenter from the same ethnic group, speaking in the appropriate language of that group, should lead to a change in response in *EP*. Neither the environment, i.e., the place of the experiment, nor the symbolic quality of the objects of the experiment should play a primordial role.

To test these three hypotheses, we conducted a similar experiment as was described by Knetsch (1989) on Kanak participants either in a Western context and in French with a Western experimenter, or in a Kanak context and in the vernacular with a Kanak experimenter in the same French environment (a vocational training center) and with French objects not typical of Kanak culture. In the first situation, in the French context, Kanak participants should behave similarly to Western participants under the same conditions. However, in the Kanak context, we should observe a decrease, or even an absence, of the endowment effect. This behavior should be similar to that of participants undergoing the experiment in a strongly Kanak place (the tribe) and with objects that are strongly prototypical of objects used during the custom ceremony.

## 3. Experiment: The Exchange Paradigm in French and Kanak Contexts

### 3.1. Materials and Methods

#### 3.1.1. Participants

Three hundred and sixty adult participants in continuing education in metropolitan France (90 participants) and New Caledonia (270 participants) were recruited^6^. The 90 participants from metropolitan France (40 women and 50 men aged 28 to 48, M = 34.6) came from urban areas in the outlying departments around Paris (Essonne, Yvelines, Haut de Seine and Val d'Oise). They were all native French speakers and natives of metropolitan France (for simplicity we will refer to them hereafter as “French participants”). All participants were normotypical and not prone to depressive disorders nor to burnout. Indeed, it was observed that there was no endowment effect in participants suffering from burnout (Jamet and Baratgin, 2018). The 270 participants (133 woman and 137 men aged 22 to 40, M = 32.6) from New Caledonia were French of Kanak origin. They all lived on the mainland, in the Northern Province, in the towns of Koumac (west coast) and its surroundings, but also in the towns of Ouéga and Poindimié and its surroundings (east coast). They all were French speakers but their mother tongue was a Kanak vernacular language, mainly the Nemi and Nixumwak languages^7^ (for simplicity we will refer to them hereafter as “Kanak participants”).

#### 3.1.2. Experimental Conditions and Materials

From the two contexts, “Western” and “Kanak,” we designed four conditions. In all conditions, the experimental procedure was exactly that of Knetsch (1989).

**Figure 1:**
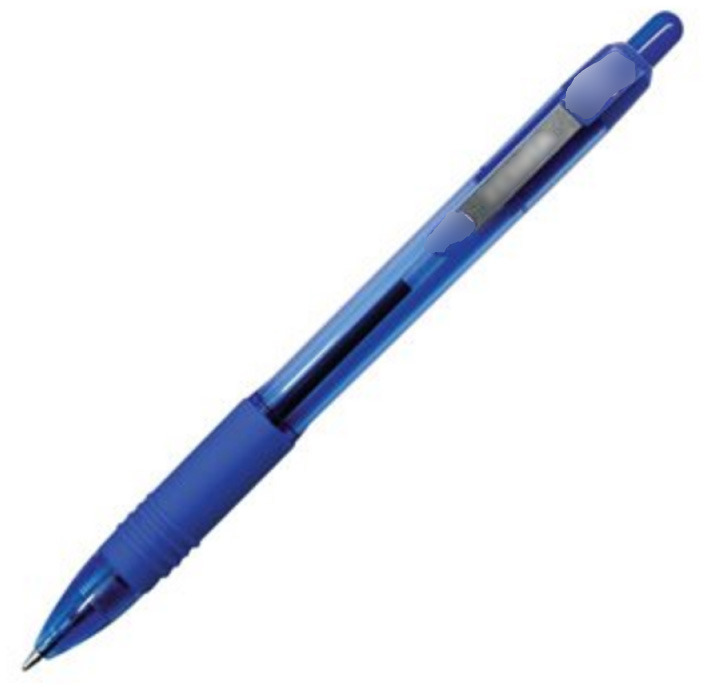
The Bic pain used for object *A* in WCF, WCK, and KCF conditions.

**Figure 2:**
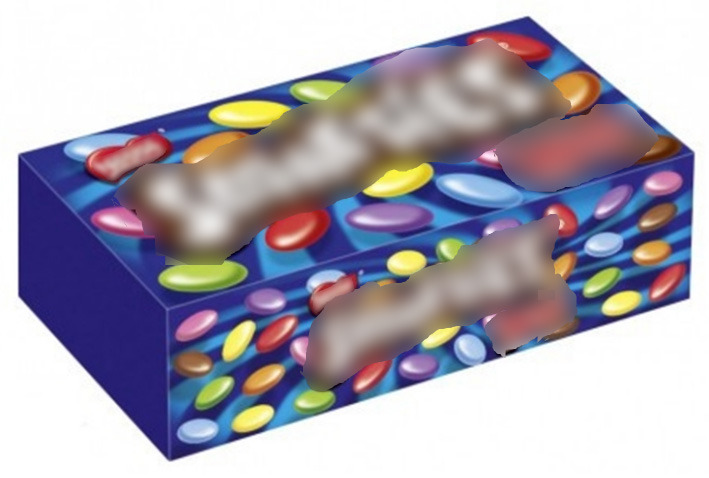
The Smarties box used for object *B* in WCF, WCK, and KCF conditions.

**Figure 3:**
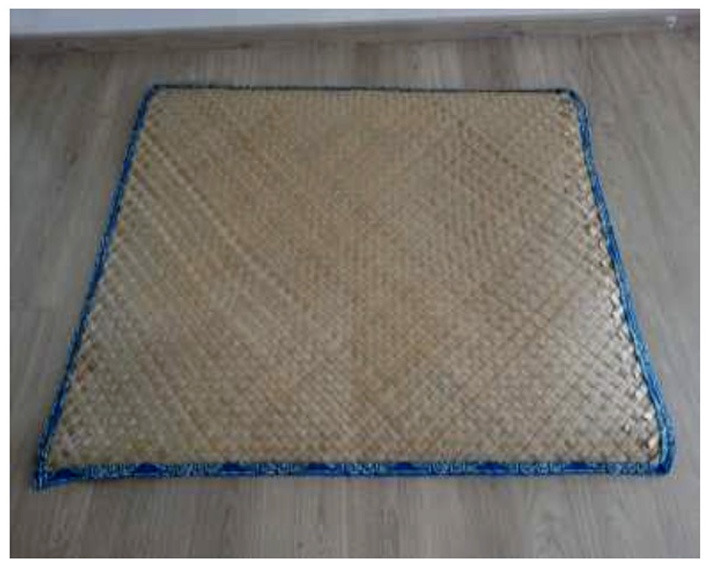
The small braided mat of Pandanus used for object *A* in the KCK condition.

**Figure 4:**
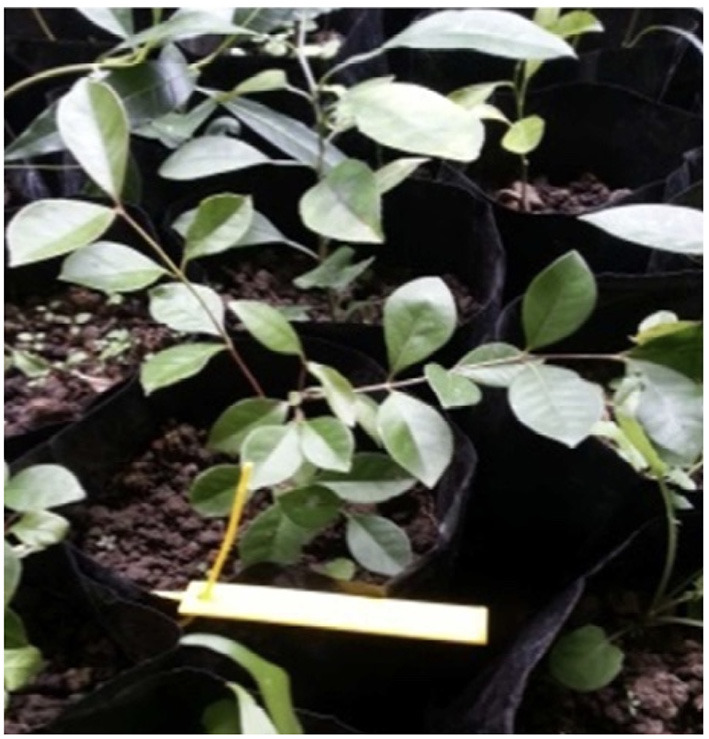
The fruit tree used for object *B* in the KCK condition.

1. The “Western” context (noted WC): the experiment took place in a French vocational training center, the experimenter was French and spoke in French. The objects were French objects: the object *A* was a Bic pen and the object B was a mini box of smarties (see Figures 1, 2). The economic value of these two objects was low. It was close to 100 Pacific francs^8^ and was 0.50 euro cents in France. This WC context was carried out with two groups:
  (a) A group of French participants (condition noted WCF, which will be our control condition),
  (b) A group of Kanak participants (condition noted WCK).

2. The “Kanak” context (noted KC) in which the experimenter was Kanak and spoke in a Kanak vernacular language, in two conditions:
  (a) In a French environment (noted KCF): the experiment was carried out in the same French vocational training center and with the same French objects as in the “Western” context (A was a Bic pen and object B was a mini box of smarties (see Figures 1, 2),
  (b) In a Kanak environment (noted KCK): the experiment took place within the tribe and the two objects were those found during the custom ceremony. Object *A* was a small braided mat measuring 1.30 × 1.50 m made of pandanus and object *B* was a fruit tree plant (see Figures 3, 4). The economic value of the two objects was similar: 1,000 Pacific francs.

The 90 French participants were all distributed in the WCF condition (control group). The 270 Kanak participants were randomly distributed according to their sex and age in one of three conditions: WCK (44 women and 46 men from 22 to 35 years old, M = 35 years old), KCF (45 women and 45 men from 22 to 40 years old, M = 32.2 years old) and KCK (44 women and 46 men from 22 to 44 years old, M = 30.6 years old). A summary of the donation design is given in Table 1 below.

**Table 1:** Design features and results for the four conditions (*N* = 360).

	**WCF**	**WCK**	**KCF**	**KCK**
	***N* = 90**	***N* = 90**	***N* = 90**	***N* = 90**
**Design**				
Participants	French	Kanak	Kanak	Kanak
Experimenter female	French	French	Kanak	Kanak
Language	French	French	Vernacular	Vernacular
Location	Vocational training center	Vocational training center	Vocational training center	Tribes
Object *A*	Bic pain	Bic pain	Bic pain	Pandanus
Object *B*	Smarties box	Smarties box	Smarties box	Fruit tree plant
**Results**				
P_Group_	*N*_*P*_ = 30	*N*_*P*_ = 30	*N*_*P*_ = 30	*N*_*P*_ = 30
Prefer object *A*	16 (54%)	15 (50%)	14 (46%)	14 (46%)
Prefer object *B*	14 (46%)	15 (50%)	16 (54%)	16 (54%)
A_Group_	*N*_*A*_ = 30	*N*_*A*_ = 30	*N*_*A*_ = 30	*N*_*A*_ = 30
Keep object *A*	26 (86%)	24 (80%)	17 (56.7%)	17 (56.7%)
Trade for object *B*	4 (14%)	6 (20%)	13 (43.3%)	13 (43.3%)
BGroup	*N*_*B*_ = 30	*N*_*B*_ = 30	*N*_*B*_ = 30	*N*_*B*_ = 30
Keep object *B*	24 (80%)	25 (83%)	13 (43.3%)	16 (54%)
Trade for object *A*	6 (20%)	5 (17%)	17 (56.7%)	14 (46%)
Difference A_Group_ (keep object *A*) − B_Group_ (trade for object *A*)	20 (66%)	19 (63%)	0 (0%)	3 (0.7%)
Z, *p*-value^a^	5.17, <0.001	4.91, <0.001	0, .50	0.77, .22

a *The null hypothesis is that the percentage of participants who chose to keep object A received in AGroup is equal to the percentage of participants who chose to exchange object B received with object A in BGroup. The alternative hypothesis is that there is an endowment effect, i.e., the percentage of participants who chose to keep object A received in AGroup is greater than the percentage of participants who chose to exchange object B received with object A in BGroup (Zellen test)*.

#### 3.1.3. Procedure

For all conditions (WCF, WCK, KCF, and KCK), data collection was carried out in several stages with groups of participants. For WCF, WCK, and KCF, it was carried out in a vocational training center during practical work, whereas for KCK the data collection took place during different visits, according to the events (births, marriages, deaths, visits) of the inhabitants, after having proceeded to a custom of “good morning.”^9^ The procedure was that of Knetsch (1989). For the four conditions the participants were randomly distributed in the three following groups:

1. P_Group_ evaluated the preference of the objects *A* and *B*. Participants were asked to choose which of object *A* or object *B* they wished to receive. To assess the preference between the two objects, the experimenter (female)^10^ presented them to the participants. She asked if they preferred object A or object B. This question was formulated in the French language by a French experimenter (female) in the WCK and WCF conditions and in the vernacular language by a Kanak experimenter in the KCF and KCK conditions.
2. A_Group_ received object *A*, and to which the experimenter (female) then asked if they wished to exchange it with object *B*. The procedure was as follows:
  (a) The experimenter handed object *A* to the participant and said: “I am giving you [Object A]. Keep it, it is yours.” This information was given in French by the French experimenter in the WCF and WCK conditions and in vernacular by the Kanak experimenter in the KCF and KCK conditions. The experimenter placed object *A* in front of each participant.
  (b) The experimenter then gave a one-page document (masking task) to each participant. The participant, after having indicated the date, their first name, their age, their place of birth, the languages spoken and their education proceeded with the task. For the French participants of WCF and Kanak participants of the WCK and KCF, a questionnaire on the professional project was proposed to them (see document to https://osf.io/4cz8y/). The activity lasted about 15–20 min. For the Kanak participants in the KCK condition, the task consisted of translating a rhyme from French into the vernacular language (see document at https://osf.io/ey829/).
  (c) Once the task was completed, the experimenter asked each participant privately whether or not they agreed to exchange object *A* for object *B*. “Earlier, I gave you this [object *A*]...” The experimenter points to object *A*. “Would you be willing to exchange your [Object *A*] for this [Object *B*]. This statement was made in the French language by the French experimenter in the WCF and WCK conditions and in the vernacular language by the Kanak experimenter in the KCF and KCK conditions.
3. B_Group_ received object *B*. The female experimenter then asked if the participant agreed to exchange it with object *A*. The procedure was otherwise the same as in A_Group_.

### 3.2. Results

The results are given in Table 1. Participants in P_Group_ for the WCF, WCK and KCF conditions showed an indifference between receiving object *A* (the Bic pen) or object *B* (the smarties box). Thus, these objects, although strongly Western, were preferred in the same way by French and Kanak participants. Similarly, the Kanak in condition KCK did not show any form of preference for object *A* (pandanus) nor for object *B* (fruit tree seedling). There was no difference compared to a random choice 50/50 (*Z* = 0.26, *p* = 0.4 for WCF, *Z* = 0, *p* = 0.5 for WCK, *Z* = −0.26, *p* = 0.6 for KCF and KCK). We analyzed the endowment effect in the exact same way as Knetsch (1989); Plott and Zeiler (2007); Knetsch and Wong (2009) by looking at whether we could observe a strong exchange asymmetry between A_Group_ and B_Group_ (*Z* = 5.17, *p* < 0.001 for WCF and *Z* = 4.91, *p* < 0.001 for WCK). As expected, we found a strong endowment effect (exchange asymmetry) in the WCF condition which matched the classical results of the standard of Knetsch (1989). We also observed a strong endowment effect in the same proposition of Kanak participants in the WCK condition. We found no difference between WCF and WCK neither for A_Group_ (26 participants kept object *A* for WCF, 24 for WCK, *Z* = 0.69, *p* = 0.24, and 24 kept object *B* in WCF, 25 in WCK, *Z* = −0.63, *p* = 0.76) and for B_Group_. We observed an absence of endowment effect in the other two conditions: KCF and KCK. The participants behaved in the same way whether they were in A_Group_ or in B_Group_. When comparing KCF and KCK, we found a similar proportion of participants who kept object *A* in A_Group_ and participants who had chosen object *A* in P_Group_ (17 vs. 14 in KCF and KCK, *Z* = 0.77, *p* = 0.22) who kept object *B* in A_Group_ and participants who chose object *B* in P_Group_ (13 vs. 16 in KCF, *Z* = −0.77, *p* = 0.78 and 14 vs. 16 in KCK, *Z* = −0.52, *p* = 0.69).

## 4. Discussion

The aim of this study was to propose a new explanation for the endowment effect observed in *EP*. The endowment effect would be due to the respect of the social norms in force in the individuals' society. Our results seem to be coherent with this hypothesis. Kanak participants are subject to the endowment effect only when the context of the experiment involves interaction with a French experimenter and in a communication made in French (WCK). The endowment effect found is comparable to the one obtained under the same conditions with French participants from metropolitan France (WCF). On the other hand, when the experiment involves an interaction between Kanak (experimenter and participants), in a communication expressed in the vernacular language of the participant, the endowment effect disappears (KCF). Acceptances and refusals of the exchange are balanced in the same proportions as the participants' preferences of the two objects. It is important to note that this change in behavior is observed while *EP* is performed with Western objects, in a Western location and without any other explicit information. The lack of an endowment effect found is comparable to the situation in which the ceremonial context of Kanak exchange is accentuated by the experimenter's words and the performance is conducted in the tribe with prototypical customary objects (KCK). These results have several important implications, not only for understanding the endowment effect found in *EP* by Knetsch (1989) but also on other aspects discussed below.

These results corroborate the criticisms of Plott and Zeiler (2007) on the traditional explanation of loss aversion (Thaler, 1980; Knetsch, 1989; Kahneman et al., 1990). But they are also in dissonance with alternative explanations, whether it be the evolutionary one of a defense of the territory (Heifetz and Segev, 2004; Huck et al., 2005) or those stemming from a particular attachment to the object (see for a review Morewedge and Giblin, 2015). Indeed, the Kanak participant does not seem to have any aversion to exchanging the object in the KCF or KCK condition and does not show any indication of a particular desire to own it. In the Western context, explanations in terms of loss aversion and territorial defense seemed relevant. In fact, our explanation was not visible because it blended with these ones, since the model of Brown and Levinson (1978, 1987) explains the refusal of the exchange with both loss aversion or defense of territory. It is the use of a different social context that distinguishes all these explanations. Our study is a new example of the importance, for cognitive psychology, to take into consideration the points of view coming from other disciplines like anthropology (Sperber and Hirschfeld, 1999). They illustrate, indeed, the ideas of Mauss (1924); Malinowski (2018) on the exchange of gifts in many traditional societies in the World, especially in Oceania.

With this experiment, we provide new experimental arguments in favor of a pragmatic explanation of the endowment effect observed in *EP*. Culture, and in particular the social norms of individuals, shapes the pragmatic interpretation of the experimenter's offer as a gift (as Plott and Zeiler, 2007, were the first to make the hypothesis). In a French context, the least onerous interpretation of this unusual action is to consider the object as a present. In the Kanak context, the interpretation of the gesture of giving is different, it is an introductory exchange in order to build or consolidate a social bond; it is a usual (even anodyne) gesture in the Kanak world. The proposal of exchange in the French context is confusing. It illustrates a violation of the social rules expected after the offering of a present. It causes an updating of the implicatures of the participant regarding their expectations of the experimenter which results in a refusal of the exchange. In the Kanak context, the experimenter's exchange proposal does not cause an update of the Kanak participant's implicatures. The latter continues to infer that the experimenter wishes to further strengthen the social bond. There is no obligation to refuse this proposal, but one can also decide that the exchange will take place later with another object (of higher value).

According to this explanation, the refusal to exchange in the French context should not be interpreted as a lack of rationality on the part of the participant. Similarly, the Kanak participant in the Kanak context, who does not produce the endowment effect, should not be interpreted as behaving more rationally. In both situations the participant's decision is consistent with their implicatures and representations of the experimenter's expectations. For participants the value of the object is not the same because the object does not have the same meaning and value. When they are asked for their preference between two equivalent materiel objects in P_Group_, the objects are simply physical objects (objet_physical_). Then, depending on the French or Kanak context, they have either the status of a present (objet_present_), or the status of an exchangeable good serving as a social link (objet_social_). They symbolically represent the gift that must continually nourish and recreate the social bond, precisely because what circulates (when and if it circulates) is the result, not the cause, of the social bond itself (Godbout, 2009). In all these situations, we are exactly as in the Bayesian situation of a different subjective probability judgment of the same event according to the context (Baratgin, 2002, 2009; Baratgin and Politzer, 2006, 2007, 2010).

A good has no “utility,” in the sense of intrinsic physical quantity, outside its relationship with an individual who desires it. The experiment underlying the definition therefore concerns individual behavior. Like any psychological notion, to have sense from an operational viewpoint, it must be defined on the basis of behavior. When we are dealing with physical quantities, the experiment is obviously made as observer independent as possible (observer dependence would be a source of error). (de Finetti, 2012, p. 262).

This observation of the variability of the status of the object by the participant according to the cultural and social context is to our knowledge a new experimental result. This result illustrates Searle and Willis (1995) definition of “social objects”: Social objects are created by the fact that we consider or count a physical object as something that goes beyond the physical structure of that object, thus conferring on it a social status in a certain context - for example, by virtue of collective recognition, a piece of paper counts as a fifty-euro bill in the context of the economy. In the Western French context of *EP*, the object is considered by the Kanak participant as object_present_ which is difficult for them to exchange, whereas it becomes object_social_, in the Kanak context [this absence of an endowment effect when the object is perceived as an exchange good is, moreover, in line with the observations of Svirsky (2014) of an absence of endowment effect for money]. The gift of the object in Kanak society (as analyzed by Mauss, 1924) can be interpreted as an *institution* (in the sense of what makes the cohesion of society as defined by Searle and Willis, 1995) resulting from the self-transcendence of the social relations that the gifts themselves are expressly designed to create and according to which individuals orient their behavior (Cedrini et al., 2020). For a renewed reading of Mauss's work on exchanges (Tcherkézoff, 2016).

Our results also offer a new example of the flexibility of bicultural individuals, observed in other contexts (Gardner, 1985; LaFromboise et al., 1993). In particular, it corroborates the results found by Chuah et al. (2007, 2009) in the ultimatum game. The study compared the decision-making behavior of participants from Malaysia and Great Britain, taking into account the location of decision-making. It was observed that the amount offered was generally higher in the Malaysian treatment group (Malaysian offerers and responders) than in the British treatment group (British offerers and responders). However, when the groups were crossed, the Malaysian proposers generally offered lower amounts to the British but not to the other Malays. The British, however, did not change their behavior.

Finally, our results argue that, contrary to the assumption of economic theory that rational agents are self-interested, individuals' decision making is strongly influenced by social interactions such as social concerns for justice, fairness, and reciprocity (Gouldner, 1960; Henrich et al., 2005; Fiddick et al., 2013; Geraci, 2020; Culpeper and Tantucci, 2021; Geraci and Franchin, 2021; Geraci et al., 2021). In particular, our work is further evidence of the need to broaden the range of regions for cross-cultural investigation for cognitive psychology and experimental economics (Henrich et al., 2010; Masuda et al., 2020). This opening should also be done for the study of populations from holistic societies, the great majority of which are from Asian countries (see for example Masuda and Nisbett, 2001; Nisbett et al., 2001; Nisbett and Masuda, 2003; Nisbett and Miyamoto, 2005; Choi et al., 2007; Yama et al., 2007; Nakamura et al., 2018).

## 5. Conclusion

The essential proposition that has been developed and tested in our study is that the answer to the offer of exchange in *EP* crucially depends on the social norms at play in the contextualized interactions between experimenter and participant. In *EP*, two key elements of social interaction shared by all human societies are brought into play: *gift* and *exchange*. This paradigm, which seems disconcertingly simple, is much more than an experimental paradigm that allows us to evaluate the endowment effect. It is a paradigm offering the possibility of understanding the core of social interactions in all human societies. In this paper, *EP* makes it possible to account experimentally, for the first time in psychology, for the particularity of exchange-based interactions between Kanak. It may also allow for the study of more detailed predictions made by anthropologists on interactions linked to filiation (Godin, 2015, 2018).

The Kanak, who are partially bicultural, show a flexibility, depending on the context, to give a response either in accordance with French social norms of politeness or in accordance with Kanak social norms of exchange (gift and return-gift). This result, however, may seem to contradict certain results in the literature. First of all, the behaviors, similar to the endowment effect, observed in certain primates (Lakshminaryanan et al., 2008; Kanngiesser et al., 2011; Brosnan et al., 2012; Flemming et al., 2012) cannot be explained in terms of the social norms of politeness (of a Western society). It is the same to explain the appetence of very young children (2 years old) to keep an object that they have just received (Gelman et al., 2012; Hood et al., 2016). Indeed the concept of property and especially that of transfer of property, are completely acquired only from 4 to 5 years old (Blake and Harris, 2009, 2011; Nancekivell et al., 2013; Davoodi et al., 2020). This is the age when children, unlike apes, respect property as a cooperative arrangement, in which they inhibit their tendency to take the property of others on condition that others do the same (Kanngiesser et al., 2020). The acquisition of social norms starts from the age of 3 years old (Schmidt et al., 2016), but the norms of politeness, seem to be acquired even later (Axia and Baroni, 1985; Baroni and Axia, 1989). One can thus probably think that the endowment effect is part of a developmental trajectory and would take two forms. The first one, “primitive,” in primates and young human primates, can be explained by an evolutionary justification (Bruner et al., 2020). The second, more sophisticated, depends on the pragmatic abilities and the capacity toward a theory of mind of the individual and manifests itself by behaving accordingly to the specific social norms of the society in which one lives. This hypothesis makes it essential to reproduce our study with Kanak children of several ages. A very recent study (Prou, 2021) indicates an endowment effect in Kanak children aged 4-5. However, this study does not allow any conclusions to be drawn because it was conducted in a French context (nursery school with a French teacher/experimenter and in French). The study carried out among older Kanak children aged 6–7, conducted in a tribe, by a Kanak experimenter and in the Kanak language, indicates a reverse endowment effect (the vast majority of children accept the exchange (Jamet et al., 2017a,b; Jamet and Baratgin, 2018). This result can be explained by the identification of children with their cultural group, which leads them to imitate in excess the behavior observed in adults. The next studies should be conducted under a comparative life span approach with the two different contexts.

## Data Availability Statement

The raw data supporting the conclusions of this article will be made available by the authors, without undue reservation.

## Ethics Statement

The studies involving human participants were reviewed and approved by Mr. Maudinet Marc, PhD in anthropology, Former pedagogical director of the master Gestion et Politiques du Handicap Sciences Politique of Paris, freelance councilor expert in the European Council, president of the scientific council of the FISAF. Mr. Deberge Dominique, retired professor of economy and management, retired education psycho-sociologist, Noumea, New Caledonia. Ms. Wanguene Marie-Louise, pedagogic councilor FELP, deputy mayor of the commune of Hienghène, Haut-Coulna tribe, New Caledonia. Mr. Lionel Zannier, manager of the formation of specialized teachers in the institute of formation of teachers of New Caledonia, Noumea, New Caledonia. The patients/participants provided their written informed consent to participate in this study.

## Author Contributions

JB and FJ: conceptual elaboration and design of the study. FJ and PG: data collection. JB: data analysis and draft of the manuscript. JB, FJ, and PG: critical revision of the manuscript. All authors contributed to the article and approved the submitted version.

## Funding

We thank the P-A-R-I-S Association for the technical and financial help we received as well as the CHArt laboratory which participated in financing the publication of the article in open access.

## Conflict of Interest

The authors declare that the research was conducted in the absence of any commercial or financial relationships that could be construed as a potential conflict of interest.

## Publisher's Note

All claims expressed in this article are solely those of the authors and do not necessarily represent those of their affiliated organizations, or those of the publisher, the editors and the reviewers. Any product that may be evaluated in this article, or claim that may be made by its manufacturer, is not guaranteed or endorsed by the publisher.

## Footnotes

^1^Recent experimental results, however, have revealed that toddlers have a very helpful behavior toward agents regarding biological needs, such as food and shelter, an effect that can also be seen with teenagers and adults (see Geraci and Franchin, 2021; Kienbaum and Mairhofer, 2021).

^2^In the Apicella et al. (2014, Appendix p. 1) describing the procedure, the context of giving a present is clearly stated: “Experiments were conducted privately, in Swahili, by one of the authors (Apicella) during her last day of residence in each camp. On this final day, gifts, such as knives and pots, are normally given to participants as an expression of gratitude. Added to the gift list were lighters and cookies. Participants were called individually to receive their gift and upon approaching the experimenter the following occurred...”.

^3^The ultimatum game (Harsanyi, 1961) illustrates the final negotiation phase between two players: a “proposer” *A* and a “responder” *B*. *A* initially receives a certain number of units (usually 10) from the experimenter. *A* must offer to give some of his units to *B*. The latter can accept or refuse their offer. If *B* accepts the offer, the units are exchanged between the two players and kept. If *B* refuses, both players lose all the units. In a second round of play, player *B* becomes the proposer and player *A* the responder. Economic theory states that it is rational for the respondent to accept all possible offers from the proposer even if they are minimal (i.e., if it is only one unit) because the alternative is not winning anything. However, the great majority of experiments on the ultimatum game has shown that minimal offers (less than 3 units) are very often rejected. Fair share offers (4, 5 units) are widely accepted (see for a recent study Beaunay et al., 2022).

^4^Several comparative studies of languages other than English have shown significant differences (see Urquizu, 2009, for a review). We take the model described by Brown and Levinson (1987) as “universal” in the sense that it provides a general framework to explain (in part) the participants' implicatures in *EP*. These implicatures of the participants, on the expectations of the experimenter, are a function of the context and of the cognitive effort that they produce (Sperber and Wilson, 1986; Escandell-Vidal, 1996; Jary, 1998). These two notions of context and cognitive effort are themselves strongly dependent on the specific social norms known by the participants. This knowledge leads to different politeness strategies (Spencer-Oatey and Jiang, 2003).

^5^In psychology or experimental economics classes, students may receive goods in exchange for their participation as participants in experiments, but this is always explained and the goods are often given after the experiment.

^6^Written informed consent to participate in this study was provided by the participants. All data was collected anonymously. The experiment was reviewed and approved by the Ethics Committee of the P-A-R-I-S Association. The Ethics statement can be obtained here: https://osf.io/6hdxs/.

^7^There are about 17 vernacular languages and 6 dialects in the Northern Province.

^8^1 Pacific franc is about 0.0083 Euro. We chose inexpensive objects, as noted above, as this avoids the refusal of the object by the Kanak participants who could not subsequently return a good of at least the same value (Henrich et al., 2005).

^9^When meeting Kanak friends, the custom is to exchange a mat, a banknote, etc.

^10^To avoid gender affecting the quality of the exchanges, the experimenter was always a female in all our groups. The Kanak experimenters collected the data within their tribe. They were known to some of the participants (those in the clan).
